# Utilization profile of emergency department by irregular migrants and hospitalization rates: lessons from a large urban medical center in Tel Aviv, Israel

**DOI:** 10.1186/s12939-020-1152-6

**Published:** 2020-04-29

**Authors:** S. Shachaf, N. Davidovitch, P. Halpern, Z. Mor

**Affiliations:** 1 Department of Health Systems Management, School of Public Health, Faculty of Health Sciences, Ben Gurion University of the Negev, POB 653, 8410501 Beer Sheva, Israel; 2 Department of Emergency Medicine, Tel Aviv Medical Center, Tel Aviv, Israel; 3 Faculty of Medicine, Tel Aviv University, Tel Aviv, Israel; 4 School of Health Sciences, Ashkelon Academic College, Ashkleon, Israel

**Keywords:** Emergency department, Health insurance, Immigration, Israel, Undocumented migrants

## Abstract

**Background:**

Irregular migrants (IMM) are excluded from the National health insurance in most developed countries and may use the emergency department (ED) as a source for medical care.

This study aims to compare the use of ED by IM with that of Israeli citizens (IC) in a large urban hospital in Tel Aviv, including socio-demographic characteristics, hospitalization proportion and medical conditions on admission.

**Methods:**

This cross-sectional study included all IM and IC patients older than 18 years who attended the ED between 2007 and 2011, and compared their socio-demographic characteristics, the administrative details of the visit and clinical variables upon admission. Hospitalization proportion was calculated by dividing the number of patients who were admitted to the hospital ward by the number of all patients who attended the ED.

**Results:**

IM who attended the ED were younger compared to IC (mean 39 ± 17 versus 52 ± 22 years, respectively), mostly males (1.4 Male/Female ratio) and mainly originated from developing countries. IM were more commonly self-referred, more likely to attend the ED during evening hours and weekends, complained of occupational injuries and frequented the surgical rather the medical ward of the ED compared with IC. IM stayed at the ED for longer periods than IC, yet the proportion of their hospitalization was lower than that of IC (19.4% versus 23.5%, respectively).

**Conclusion:**

IM stayed in the ED for longer periods and were less likely to be admitted to the hospital wards, suggesting presentation of non-severe medical conditions or possible barriers in ensuring care continuity in the community following discharge. Minimizing the barriers of IM to primary care in the community can reduce unnecessary referrals to the ED. Additionally, hospitals managements should respond to the high-volume of IM by shifting staff to busy hours and improving the communication with IM.

## Background

The World Health Organization estimated that there are ~ 214 million international migrants globally [1]. Migration flows comprise a wide range of populations, such as laborers, refugees, students and irregular migrants (IM), while each has its own medical needs and unique levels of vulnerability. IM are foreign-born persons who entered the hosting country without permission [2]. IM in many developed countries are excluded from the national health insurance and their access to healthcare is limited. Even in countries where the access to health care is guaranteed to migrants, IM may still be confronted with barriers precluding the optimized use of medical care [3]. These barriers include cultural gaps, language difficulties, different health perceptions and additional administrative and structural obstacles [4]. The limited access of IM to primary and preventive healthcare services in the community forces the migrants to seek for medical care through alternative routes, such as emergency departments (ED) for urgent and serious medical conditions, but also for non-severe medical conditions [5]. For example, IM in Spain were using ED services more commonly than Spanish born in a rate ratio of 1.4 for males and 2.2 for females [6]. In another systematic review performed in European countries, migrants were more likely to present to the ED during off-office hours and more likely than non-migrants to use the ED for low-acuity presentations [7].

The medical staff in the ER is supposed to assess the medical condition and treat IM, and also to respond to various social determinants of health influencing the medical conditions of the migrants. For example, the staff should understand the occupational exposures of the IM to certain diseases or risk for on-job injuries, and to respond to social and economic aspects related to their ability to follow the medical recommendation after discharge due to poor housing conditions and insecure employment. Additionally, hospital managements may be concerned with the limited ability of the migrants to pay for the services, especially in cases of hospitalization, as admissions are costly and are often not reimbursed by7 the IM [8]. The providers in the ED who are considering hospitalizing IM are confronted with conflicting challenges- the inadequate medical care in the community on one side, and limited ability of IM to pay for the hospital charge on the other side.

Israel absorbs all Jewish migrants under the law of return [9]. Those migrants are naturalized upon arrival and included in the national health and welfare insurance schemes form the first day. IM in Israel are not entitled for those benefits and are excluded from the Israeli national insurance law. However, based on the Patients Health Rights Law [10], they can refer to ED for urgent medical care for a fee. IM can also use two free community clinics in Tel Aviv. A pervious study reported that those clinics mainly provide primary care, but are facing difficulties to respond to patients with complex medical conditions or chronic diseases due to lack of qualified medical staff or insufficient diagnostic equipment [11].

It is estimated that more than 60,000 migrants who originated from the horn of Africa (especially Eritrea and Sudan) have entered Israel, who are mostly residing in the poorest neighborhoods of Tel Aviv [12]. The Tel Aviv Sourasky Medical Center (TASMC) is the second largest hospital in Israel and is situated in the heart of the city. It therefore provides the main medical site for emergency care for the IM.

This study aimed, for the first time in Israel, to assess the use of ED services by IM and compare the presenting medical conditions, care provided at the ED and hospitalization proportion between IM and Israeli citizens (IC). In line with previous publications from other developed countries which have exclusive national health insurance for citizens [13–17], our study hypothesis was that the proportion of hospitalization of IM would be lower than IC due to the cost of hospitalization and the self-loss of working time. The findings of this study will support the managerial level in hospitals and decision makers at the Ministry of Health in designing medical services which meet the unique needs of IM.

## Methods

This cross-sectional study included all IM and IC patients older than 18 years of age who attended the adult section of the ED at the TASMC between January 1st 2007 and December 31st 2011, during the major mmigration flux of IM into Israel. The data were available from the computerized registration files. Patients who were referred to the gynecological or the pediatric wards of the ED were excluded from this study. IM were defined as non-Israeli citizens without medical insurance, whereas IC were defined as citizens or residents who were insured by the national health insurance law, regardless their country of origin. Data collected for this study included socio-demographic characteristics (age, gender, continent or area of origin and residential district), administrative details (time of arrival and discharge from the ED, division where the patients were treated, mode of referral) and clinical variables (presenting medical condition upon admission to the ED and the outcome of the ED visit). Hospitalization proportion was calculated by dividing the number of patients who were admitted to the hospital ward by the number of all patients who attended the ED from each group. Time spent in the ED was measured from the time of registration to the time of release (whether discharge to the community or admission to one of the hospital’s wards).

### Statistical analysis

Risk factors that affected ED visits were identified by logistic regression using generalized estimating equation multivariable model, which included all the independent characteristics that were statistically in the univariate analysis. Due to the gender differences between IM and IC, a shared model was performed by matching IM and IC according to gender, age (±4 years differences) and for the year of the visit. The group matching included 28,871 pairs. The association between the visitor type (IM/IC) and the likelihood of being hospitalized from the ED was tested using conditional logistic regression.

SPSS (v 20.0) was used for the statistical analyses.

## Results

Between 2007 and 2011, 549,713 attendances were recorded at the adult ED of the TASMC, of which 528,218 (96.1%) were IC and 21,495 (3.9%) were IM. Of all the patients who visited the ED, 169,878 (30.9%) were admitted to one of the hospital’s wards: 165,697 (97.5%) were IC and 4181 (2.5%) were IM.

**Fig. 1:**
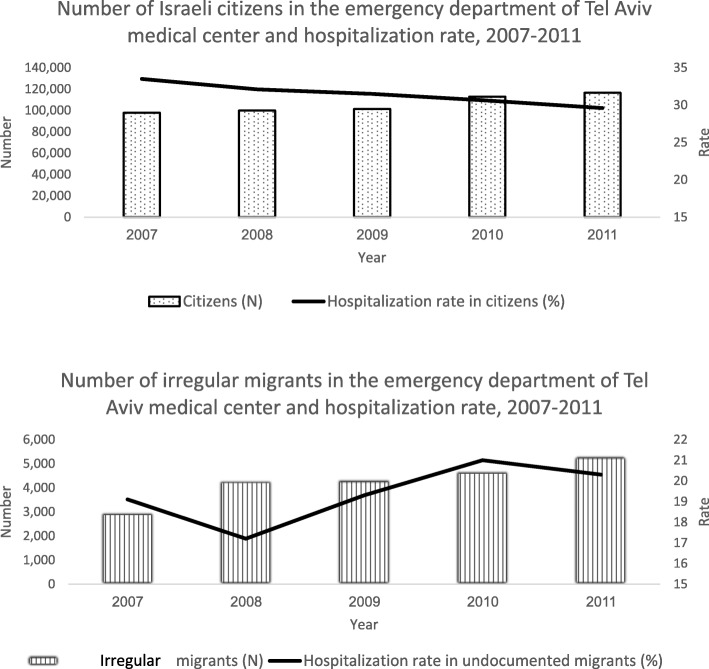
**a** Number of Israeli citizens in the emergency department of Tel Aviv medical center and hospitalization rate, 2007–2011. **1b** Number of undocumented migrants in the emergency department of Tel Aviv medical center and hospitalization rate, 2007–2011

The number of visits of IM to ED increased during the study period, yet their average hospitalization proportion was significantly lower than that of IC (19.4% versus 23.5%, respectively*, p* < 0.001, Fig. 1a and b). IM who visited the ED were younger than IC and their mean age was 39 years (SD = 17) compared with 52 years (SD = 22) for IC. The majority of IM who visited the ED were males, who originated from horn of Africa and resided in Tel Aviv (Table 1).

**Table 1:** Demographic characteristics of patients visited the emergency department by civil status, 2007–2011

Visitor type	Irregular migrants ***N*** = 21,495 (%)		Israeli Citizens ***N*** = 528,218 (%)	
Characteristic				
	Male	Female	Male	Female
Age groups (years)				
18–34	6839 (54.4)	4313 (48.3)	79,752 (30.0)	77,224 (29.5)
35–49	3127 (24.9)	2038 (22.8)	57,833 (21.7)	44,811 (17.1)
50–64	1440 (11.5)	1482 (16.6)	53,063 (20.0)	48,735 (18.6)
65+	1161 (9.2)	1095 (12.3)	75,564 (28.3)	91,236 (34.8)
Male: female ratio				
18–34	1.6		1.0	
35–49	1.5		1.3	
50–64	1.0		1.1	
65+	1.1		0.8	
Overall	1.4		1.0	
Area of origin				
Asia and Oceania	536 (4.3)	825 (9.2)	2488 (0.9)	3044 (1.2)
East Europe	494 (3.9)	572 (6.4)	58,763 (22.1)	69,726 (26.6)
West Europe	637 (5.0)	552 (6.2)	7710 (2.9)	9800 (3.7)
North Africa and the Middle East	162 (1.3)	61 (0.7)	31,112 (11.7)	32,147 (12.3)
Horn of Africa	2916 (23.2)	743 (8.3)	1409 (0.5)	1030 (0.4)
Africa, south of the Sahara	446 (3.5)	259 (2.9)	852 (0.3)	790 (0.3)
America	486 (3.9)	476 (5.3)	5676 (2.1)	6701 (2.6)
Israel	–	–	155,787 (58.5)	136,754 (52.2)
Unregistered	6890 (54.8)	5440 (60.9)	2415 (0.9)	2014 (0.7)
Residential district				
Northern Israel	104 (0.8)	53 (0.6)	12,264 (4.6)	7468 (2.9)
Jerusalem	135 (1.1)	111 (1.2)	4898 (1.8)	3213 (1.2)
Tel Aviv	9718 (77.3)	7523 (84.3)	208,394 (78.3)	215,456 (82.2)
Central Israel	465 (3.7)	295 (3.3)	31,998 (12)	28,644 (10.9)
Southern Israel	195 (1.6)	87 (1)	8111 (3)	6777 (2.6)
Unknown	1950 (15.5)	859 (9.6)	547 (0.2)	448 (0.2)

IM were more likely to be treated in the surgical ward of the ED than IC, more commonly attended the ED during the evening shifts and weekends (Fridays and Saturdays in Israel) (Table 2). Most IM self-referred themselves to the ED, and were more likely to complain of occupational injuries and violence than IC (28.8% vs. 18.8, and 4.8% vs. 1.1%, respectively). However, in cases IM were referred to the ER by health providers, they were more likely to be hospitalized than IC. IM also stayed in the ED for longer periods, regardless whether they were hospitalized or discharged to the community.

**Table 2:** Administrative characteristics of patients visited the emergency department by civil status, 2007–2011

Characteristic	Irregular migrants *N* = 21,495 (%)	Israeli Citizens *N* = 528,218 (%)
ED division		
Internal medicine	8835 (41.1)	244,737 (46.3)
Surgical	12,660 (58.9)	283,481 (53.7)
Shift		
Morning (7:00 AM - 3:00 PM)	8632 (40.2)	240,873 (45.6)
Evening (3:00 PM - 11:00 PM)	9051 (42.1)	204,080 (38.6)
Night (11:00 PM - 7:00 AM)	3812 (17.7)	83,265 (15.8)
Day of the week		
Sunday	3585 (16.7)	90,356 (17.1)
Monday	3120 (14.5)	79,627 (15.1)
Tuesday	3066 (14.3)	78,681 (14.9)
Wednesday	3075 (14.3)	76,799 (14.5)
Thursday	3026 (14.1)	79,803 (15.1)
Friday	2832 (13.2)	66,244 (12.5)
Saturday	2791 (13.0)	56,708 (10.7)
Mode of referral		
Self-referral	13,777 (64.1)	203,699 (38.6)
Health institute	7447 (34.6)	321,964 (61)
Employer	271 (1.3)	2555 (0.5)
Patient complaint		
Illness	14,491 (67.6)	381,634 (72.2)
Road accident	578 (2.7)	30,716 (5.8)
Occupational accident	5108 (23.8)	98,927 (18.8)
Violence	1040 (4.8)	5954 (1.1)
Unknown	235 (1.1)	10,987 (2.1)
Length of stay in ED (average hours ± Standard deviation)		
Visits ended with discharge from the ED		
Male	3.04 ± 2.36	2.87 ± 2.23
Female	3.14 ± 2.41	3.03 ± 2.28
Visits ended with hospital admission		
Male	2.95 ± 2.21	2.50 ± 1.87
Female	3.14 ± 2.34	2.62 ± 1.93

**Fig. 2:**
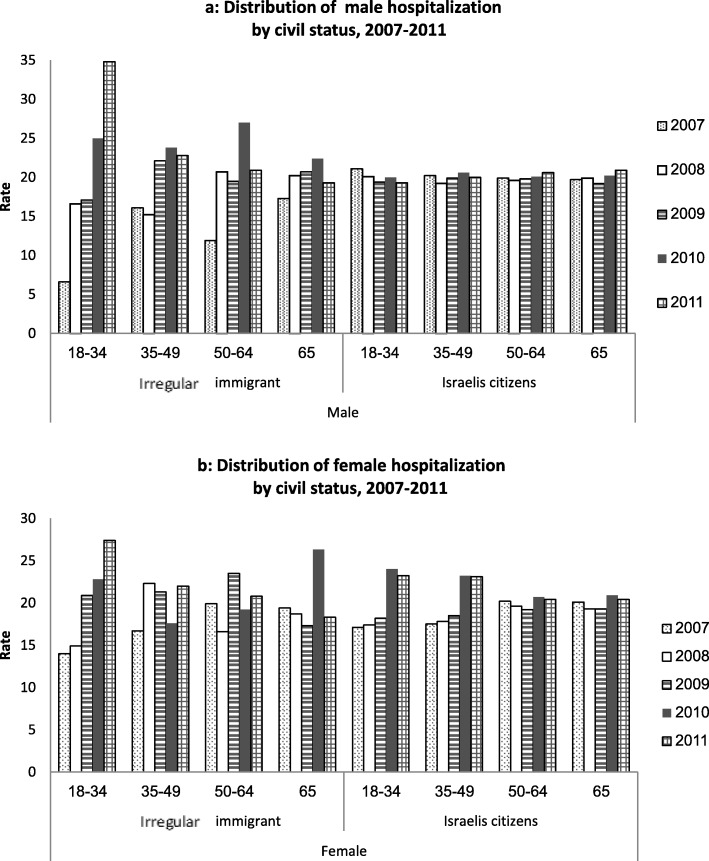
**a**: Distribution of male hospitalization rate by civil status, 2007–2011. **b**: Distribution of female hospitalization rate by civil status, 2007–2011

Male IM were more likely to be hospitalized than females (61.9% versus 38.1% of the hospitalized IM, respectively, *p* < 0.001), whereas the gender ratio of hospitalization of IC was similar for both genders (51.1% versus 48.9%, respectively, *p* = 0.1). The proportion of hospitalization of the IM generally increased with age, whereas the rate among IC was stable throughout the different age groups (Fig. 2).

Factors which increased the likelihood of hospitalization among IM included being male, older age, living outside of the city of Tel Aviv, being treated at the internal ward of the ED, attending the ED between 2009 and 2011, arriving during the evening shift and on working days and being referred by a health provider- generally similar to IC (Table 3). Logistic regression analysis adjusted for the origin of the patient, the treating ward in the ED, the daily shift, mode of referral, patient’s complaint and length of stay demonstrated that IM were less likely to be hospitalized than IC (Table 4).

**Table 3:** Factors associated with patient hospitalization from emergency department – generalized estimating equations (GEE)

		Irregular migrants *N* = 21,495 (%)		Israeli Citizens *N* = 528,218 (%)	
Characteristic	**Categories**	**OR (95% CI)**	***P*-Value**	**OR (95% CI)**	**P-Value**
Gender	Male	1.4 (1.2–1.5)	*P* < 0.001	1.5 (1.4–1.6)	*P* < 0.001
	Female	1		1	
Age (year)		1.1 (1.1–1.2)	*P* < 0.001	1.0 (1.0–1.0)	*P* < 0.001
Residential district	Other than Tel Aviv	1.4 (1.3–1.5)	*P* < 0.001	1.4 (1.2–1.7)	*P* < 0.001
	Tel Aviv	1		1	
ED division	Internal medicine	1.5 (1.3–1.6)	*P* < 0.001	1.8 (1.6–1.8)	*P* < 0.001
	Surgical	1		1	
Year of the visit	2007–2008	1.05 (1.0–1.1)	*P* < 0.004	0.9 (0.9–1.0)	*P* < 0.001
	2009–2011	1		1	
Shift	Evening	1.1 (1.0–1.3)	*P* < 0.005	1.0 (1.0–1.0)	*P* < 0.006
	Night	1.0 (0.9–1.2)	*P* < 0.661	0.9 (0.9–1.0)	P < 0.001
	Morning	1		1	
Day of the week	Sunday	1.1 (1.05–1.2)	*P* < 0.045	1.2 (1.1–1.2)	*P* < 0.000
	Monday-Thursday	1.1 (1.04–1.2)	*P* < 0.043	1.1 (1.1–1.2)	P < 0.001
	Friday-Saturday	1		1	
Mode of referral	Health institute	2.5 (2.2–2.7)	P < 0.001	1.5 (1.4–1.6)	*P* < 0.001
	Employer	0.2 (0.1–0.6)	*P* < 0.005	0.4 (0.4–0.5)	*P* < 0.001
	Self-referral	1		1	
Patient complaint	Violence	0.6 (0.4–0.9)	*P* < 0.004	0.9 (0.8–1.0)	*P* < 0.003
	Industrial accident	0.4 (0.3–0.5)	*P* < 0.001	0.3 (0.2–0.3)	*P* < 0.001
	Road accident	0.5 (0.3–0.9)	*P* < 0.009	0.3 (0.3–0.3)	P < 0.001
	Unknown	2.7 (2.0–3.8)	*P* < 0.001	1.3 (1.2–1.4)	*P* < 0.001
	Illness	1		1	
Average length of stay in ED until discharge or hospitalization (hour)		0.9 (0.9–0.9)	*P* < 0.013	0.9(0.8–0.8)	*P* < 0.001

**Table 4:** Factors influencing patient hospitalization from the emergency department – conditional logistic regression

Characteristic	Categories	OR (95% CI)	***P***-Value
Visitor type	Irregular migrants	0.8 (0.7–0.9)	*P* < 0.001
	Israeli citizens	1	
ED division	Internal	1.4 (1.3–1.5)	*P* < 0.001
	Surgical	1	
Shift	Evening	0.9 (0.9–1.0)	N.S
	Night	0.9 (0.8–0.9)	*P* < 0.000
	Morning	1	
Day of the week	Sun	1.1 (1.0–1.2)	*P* < 0.005
	Mon-Thu	1.1 (1.0–1.2)	*P* < 0.002
	Fri-Sat	1	
Mode of referral	Health institute	1.7 (1.6–1.8)	*P* < 0.001
	Employer	0.5 (0.3–0.8)	*P* < 0.008
	Self-referral	1	
Patient complaint	Violence	0.7 (0.6–0.9)	*P* < 0.003
	Industrial accident	0.3 (0.3–0.4)	*P* < 0.001
	Road accident	0.2 (0.2–0.3)	*P* < 0.001
	Unknown	1.6 (0.9–2.8)	*P* < 0.066
	Illness	1	
Length of stay (hour)		0.9 (0.9–1.0)	*P* < 0.001

The mean duration of hospitalization for IM aged 18–49 years was longer by one day and 14 h than that of that of IC in similar ages.

## Discussion

IM comprised ~ 4% of all individuals who visited the ED of the TASMC and 2.5% of its hospitalized patients. IM stayed in the ED for a longer time than IC before being discharged, yet their hospitalization proportion was lower.

The ED functions as a medical site for urgent and severe medical conditions, such as life-threatening events and other major injuries. However, because IM have limited access to ambulatory treatment alternatives, they may refer themselves to the ED also for non-severe medical conditions. This high volume of IM self-referral is likely to reflect a limited access to primary care, possible inadequate orientation of the alternative ambulatory treatment sites, dissatisfaction with previous providers in the community and the anticipation for rapid diagnosis and treatment. Additionally, language barriers, financial constraints, health literacy and lack of understanding of the healthcare system may inhibit IM to use community services [7]. The ED management is facing an increased demand of individuals who seek for medical care in the ED, while balancing between the hospitals’ responsibility to provide emergency care and at the same time to serve as a “safety medical net” for IM who can not get medical services in the community [18].

As expected, IM in our study were younger than IC, mostly in their working years and the majority were males. Evening hours and weekends were the preferred times for IM to attend the ED. This may be related to their work duties during the week, while trying not to miss paid days, thus using off-office hours to seek for medical services at the ED. Contrarily, IC enjoy labor rights and are eligible to take a paid sick leave and visit their medical providers during working hours. The relatively lower proportion of hospitalization of the IM compared with that of IC may suggest that their presenting medical conditions in the ER were milder and did not require intense care. This may be aligned with their younger age compared to IC. It is also possible that IM were hospitalized due to financial difficulties, as they are supposed to pay for the hospital care. Migrants may also refrain from hospitalization as they are missing working days which affect their income. Better access of IM to ambulatory care with more flexible hours may have reduced the volume of cases who attend the ER. The estimated male:female IM ratio in Israel is 5:1 [19], which may explain the lower volume of women referred to the ER. However, females’ hospitalization rate in our study was lower than that of males. This might reflect employment instability of women IM compared with that of men, and also be related to gender family commitments which resulted in limited time and opportunities for hospitalization [20].

IM complained of more occupational injuries than IC, and consequently were treated in the surgical ward of the ED. IM are generally more likely to work in jobs that expose the workers to risks [21], which are also referred as “3-D jobs” (Dirty, Dangerous, Demanding) [22]. Moreover, their employment is mostly “off-the-books”, without sufficient training and tutelage work safety.

The total length of the visit of IM in the ED was longer than that of IC. This may be of particular significance, as ED are generally overcrowded [23]. It is possible that medical staff were lacking previous medical history of IM, faced language and communication barriers with the patients and were confronted with different cultural perspective regarding health and illness [24]. The lack of previous medical information might have led to longer time spent in the ED and the need to rely on diagnostic tests, which prolonged the time required for physicians’ decision [25]. It also may be that physicians in the ED who initially planned to admit the patient in the hospital postponed the transfer, while trying to stabilize the medical condition. If the patient’s medical condition had been stabilized in the ED, she/he could have been discharged to the community, sparing the hospitalization costs. We further calculated that during the study period, all the IM stayed in the ED for a total of 2397 h, which is equivalent to 40 additional hours a month.

The volume of IM who use the ED, especially for routine non-severe medical conditions presents a unique challenges to the hospitals’ administrations, as found in other developed countries [13–17, 26]. The medical staff at the ED have to function under additional pressure, while increasing the possibility for clinical errors to occur and reducing patients’ level of satisfaction [27]. Furthermore, ED visits are costly and in cases the IM is unable to pay for the service, the hospital is not reimbursed. A financial estimation which was performed in the TASMC in 2012 found that US $5,670,103 of accumulated IM debts from unpaid ED visits and hospitalizations were not collected [28].

This is the first study performed in Israel to assess the use of ED by IM, yet it is subject to several limitations. First, the data were originally collected for administrative purposes up to 2011, and therefore specific health determinants, such as profession, education, income, living condition and complete medical records were not included. Second, IM do not have identification numbers and their names are sometime difficult to spell by the local staff. It was therefore difficult to define recurrent visits with first referral to the ED. Third, IM are usually generally younger than the general population in the hosting country and therefore are exposed to different risk and have other medical conditions. Lastly, female IM can also be hospitalized after visiting the gynecology ED, which was not captured in this study. It is therefore likely that actual female hospitalization rate was higher.

The results of this study may be used by hospitals management to improve the medical services in the ED in order to meet the migrants’ health needs, as was developed in Geneva, where a “migrant friendly” institutional culture was embedded in the hospital operational scheme [29]. For example, using a triage nurse with ability to speak several languages in order to perform a rapid clinical classification of the visits and to recommend the IM with mild medical conditions other treatment alternatives in the community. A translator in the ED may assist the medical staff in taking patients’ history and providing recommendations. Better staffing of the ED, especially in the surgical ward of the ED and on evening shifts and weekends may reduce the overload of the medical staff at the ED. On the national level, decision makers should consider granting the IM a “social residency” [30], which is unrelated to their legal status, yet entitle them to health services. This way, they can use more services in the community, precluding unnecessary visits to the ED.

## Conclusions

The hospitalization rates of IM in Israel were lower than that of citizens, and they may use the ED for non-severe medical conditions that could have been cared for in the community. In order to address the medical needs of the IM who are excluded from the national medical insurance, the access of the migrants to ambulatory community care should be improved.

## Data Availability

The datasets used and/or analysed during the current study are available from the corresponding author on reasonable request.

## Abbreviations

ED: Emergency department
IC: Israeli citizen
IM: Irregular migrant
TASMC: The Tel Aviv Sourasky Medical Center
